# Molecular mechanisms governing aquaporin relocalisation

**DOI:** 10.1016/j.bbamem.2021.183853

**Published:** 2022-04-01

**Authors:** Andrea Markou, Lucas Unger, Mohammed Abir-Awan, Ahmed Saadallah, Andrea Halsey, Zita Balklava, Matthew Conner, Susanna Törnroth-Horsefield, Stuart D. Greenhill, Alex Conner, Roslyn M. Bill, Mootaz M. Salman, Philip Kitchen

**Affiliations:** a College of Health and Life Sciences, Aston University, Aston Triangle, Birmingham B4 7ET, UK; b MRC Institute of Metabolic Science, University of Cambridge, Cambridge CB2 0QQ, UK; c School of Sciences, Research Institute in Healthcare Science, University of Wolverhampton, Wolverhampton WV1 1LY, UK; d Department of Biochemistry and Structural Biology, Lund University, PO Box 124, 221 00 Lund, Sweden; e Institute of Clinical Sciences, College of Medical and Dental Sciences, University of Birmingham, Edgbaston, Birmingham B15 2TT, UK; f Department of Physiology Anatomy and Genetics, University of Oxford, Oxford OX1 3QX, UK; g Oxford Parkinson's Disease Centre, University of Oxford, South Parks Road, Oxford OX1 3QX, UK

**Keywords:** Aquaporin, AQP, Osmosis, Membrane trafficking

## Abstract

The aquaporins (AQPs) form a family of integral membrane proteins that facilitate the movement of water across biological membrane by osmosis, as well as facilitating the diffusion of small polar solutes. AQPs have been recognised as drug targets for a variety of disorders associated with disrupted water or solute transport, including brain oedema following stroke or trauma, epilepsy, cancer cell migration and tumour angiogenesis, metabolic disorders, and inflammation. Despite this, drug discovery for AQPs has made little progress due to a lack of reproducible high-throughput assays and difficulties with the druggability of AQP proteins. However, recent studies have suggested that targetting the trafficking of AQP proteins to the plasma membrane is a viable alternative drug target to direct inhibition of the water-conducting pore. Here we review the literature on the trafficking of mammalian AQPs with a view to highlighting potential new drug targets for a variety of conditions associated with disrupted water and solute homeostasis.

## Highlights

• Aquaporins (AQPs) form water and solute permeable pores in biological membranes.
• AQPs represent attractive drug targets for disorders of water/solute homeostasis.
• Drug discovery efforts towards pore-blocking AQP inhibitors have made little progress.
• AQPs are dynamically relocalised between intracellular vesicles and plasma membrane.
• This relocalisation represents a new target for inhibition of AQP function.

## Graphical abstract

**Figure:**
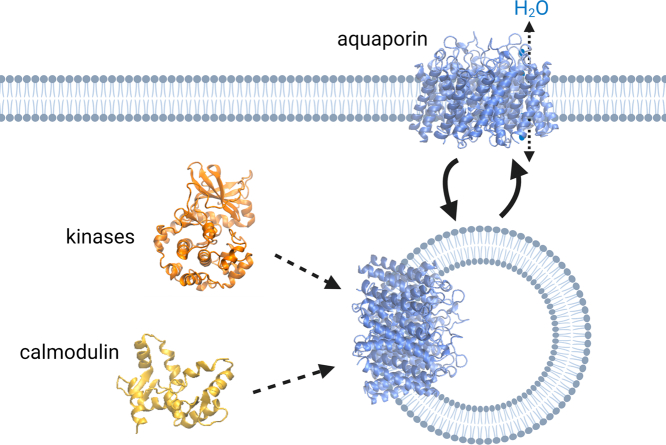

## 1 Introduction

The human body is approximately 60% water. The water content of its different compartments is tightly and dynamically regulated, from the approximately five litres of blood down to the picolitre volumes of single cells. Aquaporins (AQPs) form a family of small integral membrane proteins that facilitate the passive transport of water across all biological membranes down osmotic or hydrostatic pressure gradients. A subset of AQPs (known as aquaglyceroporins) can also facilitate the passive transport of small uncharged solutes (such as glycerol, urea, ammonia, and hydrogen peroxide). Although AQPs increase the water permeability of the membranes in which they reside, most membranes have some level of intrinsic, AQP-independent water permeability. For some AQP-transported solutes there are also AQP-independent pathways (for example UT-A and UT-B transporters provide an AQP-independent pathway for transmembrane urea diffusion [1]). In humans, there are 13 family members, at least one of which is expressed in almost all tissues. AQPs support a variety of physiological processes, including whole-body water homeostasis via the kidneys [2], cerebrospinal fluid (CSF) homeostasis [3], glymphatic system function [4], and cycling of triglyceride-derived glycerol between fat and the liver [5]. The pathophysiological consequences of AQP dysregulation include brain and spinal cord swelling following traumatic injury [6], [7] or stroke [8], cancer cell migration [9], and nephrogenic diabetes insipidus [10], and they are also implicated in neurodegenerative diseases [11] and epilepsy [12], [13].

The structural biology of the AQP family is well-established. AQPs are multipass transmembrane proteins, consisting of approximately 300 amino acid residues with predicted molecular weights for the human proteins ranging from 27 to 37 kDa. They have six transmembrane (TM) domains that are linked by five loops. Two loops consist of short half-helix membrane-embedded segments that enter and exit from the same side of the membrane, and contain the family's conserved signature Asn-Pro-Ala (NPA) motifs that form hydrogen bonds with water molecules in the pore. AQPs are homotetrameric, but each monomer functions independently as a water pore [14]. Water molecules traverse the AQP pore in single file [15]. The structure of the termini, which are cytoplasmic, is less well-understood as they are usually not resolved in crystallographicl studies due to disorder or truncation during construct design.

**Fig. 1:**
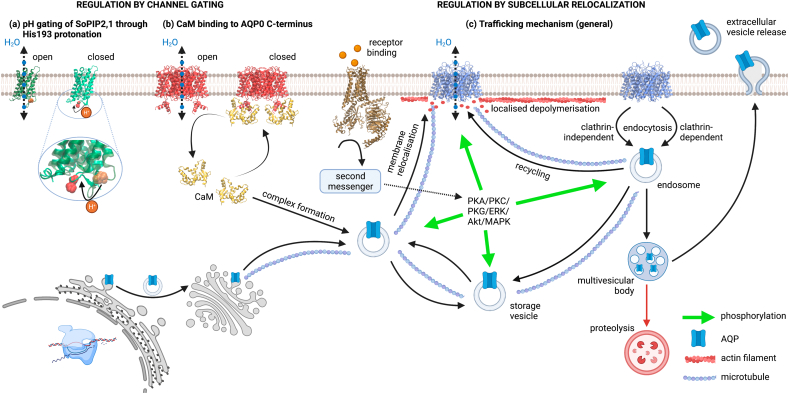
Schematic overview of aquaporin regulation. Some plant AQPs are regulated by gating such as (a) SoPIP2;1 where low pH causes protonation of His193 leading to conformational changes and pore occlusion on the intracellular side (open structure at pH 8 PDB ID: 2B5F, closed at pH 6 PDB ID: 4IA4). However, gating has only been reported for (b) mammalian AQP0 through calmodulin binding (PDB 3J41), which occludes the pore. The main regulatory mechanism for mammalian AQPs (represented by AQP4, PDB ID: 3GD8) is (c) via trafficking to and from the plasma membrane. After protein biosynthesis, AQPs are inserted into intracellular vesicles which transfer them to the plasma membrane. A population of intracellular vesicular pools also remains in the cytoplasm, which can be triggered to increase AQP membrane abundance, typically via calmodulin- and phosphorylation-dependent mechanisms. Triggers can be hormonal (e.g. vasopressin-induced localisation of AQP2 to the apical membrane of kidney collecting duct cells, V2R PDB ID: 7KH0) or environmental (e.g. hypoxia-induced localisation of AQP4 to the astrocyte plasma membrane). Internalisation through clathrin-dependent and clathrin-independent (such as caveolae) pathways reduce cell surface abundance via incompletely understood pathways. In addition, some AQPs are released into the extra-cellular space in extracellular vesicles.

There are several ways in which mammalian AQPs could be regulated to support dynamic fluid homeostasis. Changes in the rate of transcription of AQP genes can lead to changes in protein levels and therefore changes in membrane permeability. This is relatively slow (hours) as the gene must be transcribed, and the protein translated and trafficked from the endoplasmic reticulum membrane, through the Golgi, and to the plasma membrane. Changes in subcellular localisation of existing protein can be much faster. This can be mediated by changes in the rates of internalisation by endocytosis and return through recycling endosomes, or by exocytosis of dedicated “storage” vesicles (as for the well-characterised trafficking of the glucose transporter GLUT4 in response to insulin [16]). Membrane trafficking can lead to changes in the amount of AQP protein in the plasma membrane and therefore changes in membrane water permeability. Finally, changes in the single channel permeability as a result of post-translational modification (e.g. protein phosphorylation), or protein-protein interaction can lead to changes in overall membrane permeability (known as “gating”). Whilst AQP gating is well-established for plant AQPs [17], and the structural biology is well-characterised [18], in mammals, regulation of AQPs by changes in subcellular localisation is more common (Fig. 1). In this review, we focus on the rapid changes in mammalian AQP function mediated by subcellular relocalisation.

### 1.1 AQP0

AQP0, also known as major intrinsic protein (MIP), is expressed in the fibre cells of the ocular lens. Unlike other mammalian aquaporins for which regulation by gating remain controversial, direct gating of the water channel has been described for AQP0. The single-channel water permeability of AQP0 expressed in *Xenopus laevis* oocytes increased approximately two-fold in response to decreased pH. Pre-treatment with diethyl pyrocarbonate (DEPC), which covalently modifies histidine residues leaving them non-protonatable, or mutation of the external His40 residue abolished the pH response, suggesting that direct protonation of His40 mediates this increased water permeability. Removal of extracellular calcium increased water permeability of AQP0 approximately four-fold. This Ca^2+^ sensitivity was demonstrated to be dependent on calmodulin (CaM) with CaM inhibitors (trifluoperazine, calmidazolium, and N-(6-aminohexyl)-5-chloro-1-naphthalenesulfonamide (W-7)) restoring water permeability [19]. Nuclear magnetic resonance (NMR) spectroscopy was used to show that CaM binds the C-terminus of AQP0 in a calcium ion-dependent manner [20]. Further structural biology and molecular simulation studies [21] suggested a model whereby Ca^2+^-bound CaM binds AQP0 with a 1:2 stoichiometry (i.e. 2 CaM molecules per AQP0 tetramer) allosterically inhibiting water permeability. Mutagenesis of the AQP0 C-terminus confirmed that the AQP0-CaM interaction inhibited AQP0 function [22]. Phosphorylation of an AQP0 C-terminal peptide abolished CaM binding in vitro, suggesting that phosphorylation may regulate water channel activity by altering CaM affinity. Specifically, phosphorylation of Ser235, a consensus site for PKA has been shown to relieve this inhibition by altering the CaM-AQP0 interaction surfaces [23]. Moreover, the PKA-anchoring protein AKAP2 increased phosphorylation efficiency, binding AQP0 at residues 238–246 [24].

Regulation of AQP0 by subcellular relocalisation has not been described. This is likely due to the unique cell biology of the lens fibre cells whereby they progressively lose their organelles as they mature [25]. However, treatment of rat lens epithelial explants with the PKC inhibitor Go6979 prevented exit of AQP0 from the Golgi, and Ser235Ala mutation prevented Golgi exit of mouse AQP0 overexpressed in the rabbit kidney cell line RK13 [26], suggesting that phosphorylation of AQP0 is required for correct trafficking to the plasma membrane immediately following biosynthesis.

Overall, AQP0 is regulated by pH-, phosphorylation- and calmodulin-dependent gating. There is no evidence yet to suggest that AQP0 is regulated by subcellular relocalization (Table 1).

**Table 1:** Summary of the regulation of mammalian aquaporins. PKA – protein kinase A, PKC – protein kinase C, PKG – proten kinase G, ERK – extracellular signal-regulated kinase, MAPK – mitogen-activated protein kinase, PI3K – phosphatidylinositol 3-kinase, TRP – transient receptor potential.

AQP	Regulation	Trigger(s)	Mechanism(s)
AQP0	Gating	pH	PKA, PKC, calmodulin, Ca^2+^
AQP1	Trafficking, gating	secretin, hypotonicity, hypertonicity	PKA, PKC, cGMP, cAMP, Ca^2+^, calmodulin, actin, microtubules, TRP channels
AQP2	Trafficking	vasopressin, hypertonicity	PKA, AKAPs, actin, TM5b, Arp2/3
AQP3	Trafficking	adrenaline, hypotonicity, hypertonicity	PKA, PKC, RalA, PI3K/Akt/mTOR
AQP4	Trafficking	vasopressin, histamine, glutamate, hypotonicity, hypoxia	PKA, PKC, PKG, ERK, p38-MAPK, actin, TRP channels, Ca^2+^, calmodulin
AQP5	Trafficking	adrenaline, acetylcholine, hypotonicity	PKA, PKG, cAMP, Ca^2+^, actin, microtubules, TRP channels
AQP6	Gating	pH	–
AQP7	Trafficking	lipolytic stimuli	PKA, PLIN1
AQP8	Trafficking	glucagon	cAMP, PKA, PI3K, microtubules
AQP9	Trafficking	unknown	PKA, PKC
AQP10	Trafficking	lipogenic stimuli, lipolytic stimuli	unknown
AQP11	Trafficking	unknown	unknown
AQP12	Trafficking	unknown	unknown

### 1.2 AQP1

AQP1 is predominantly expressed in the brain, kidney, eye, lung, muscle, and erythrocytes [27], [28]. AQP1 was the first water channel to be isolated, in 1992 [29], although earlier work by Benga et al. had demonstrated the presence of this water channel protein in erythrocyte membranes [30].

As well as water transport, AQP1 has been suggested to act as a cGMP-gated ion channel that transports Cs^+^, Na^+^ and K^+^ [28]. The intracellular loop D was proposed to interact directly with cGMP [31] and further investigation suggested that the phosphorylation of Tyr253 in the C-terminus regulates the ion channel of AQP1 and its response to cGMP [32]. More recently, mutagenesis of the intracellular loop D suggested that this region was crucial for the binding of cGMP [33]. Our own recent work showed that interactions in this loop are crucial for the stability of the AQP1 tetramer [34].

Protein kinase C (PKC) has been implicated in the regulation of AQP1. In *Xenopus* oocytes, AQP1-dependent water permeability was markedly increased following PKC activation, whereas mutants lacking threonine 157 (Thr157Ala), threonine 239 (Thr239Ala) or both, had a reduced response or did not respond at all to PKC activation, respectively [35]. However, this study did not quantify the plasma membrane localisation of AQP1, and so could not distinguish between AQP1 relocalisation and changes in single channel permeability. In HEK293 cells, AQP1 translocated from cytoplasmic vesicles to the plasma membrane in response to extracellular hypotonicity and vice versa when returned to isotonic conditions. Microtubule depolymerisation with demecolcine blocked AQP1 relocalisation, whereas actin depolymerisation by cytochalasin D did not [36]. This translocation was blocked by inhibition of transient receptor potential (TRP) channels, removal of extracellular calcium and inhibition of CaM, as well as by either inhibiting PKC or mutating the putative phosphorylation sites Thr157 and Thr239 [37].

AQP1 localisation may also be regulated by PKA. Purified PKA was shown to phosphorylate immunoprecipitated AQP1 in vitro, and activation of PKA by cAMP analogues and forskolin increased the AQP1 abundance at the plasma membrane of *Xenopus* oocytes [38]. In primary rat cholangiocytes, stimulation with the peptide hormone secretin (which signals via cAMP in cholangiocytes [39]) led to increased endogenous AQP1 in the plasma membrane fraction following subcellular membrane fractionation by sucrose density gradient centrifugation, as well as increased plasma membrane water permeability [40]. In mouse cholangiocytes, transfected rat AQP1-GFP relocalised to the apical membrane in response to secretin or dibutyryl-cAMP [41]. However, PKA inhibition did not inhibit the hypotonicity-induced relocalisation of human AQP1-GFP in HEK293 cells [36], suggesting that PKA regulation of AQP1 localization may vary between cell types and species.

CaM inhibition by W-7 or removal of extracellular calcium prevented AQP1-GFP relocalisation in HEK293 cells [37], and W-7 inhibited changes in GFP-AQP1-mediated water permeability in transfected human umbilical vein endothelial cells (HUVECs) [42], although the mechanism by which CaM might modulate AQP1 remains to be explored.

Overall, the subcellular localization of AQP1 is controlled by phosphorylation via at least two different protein kinases (PKC and PKA); this regulation may be cell-type specific. AQP1 is further indirectly regulated via activation of TRP channels, and may be controlled by CaM via an as-yet-undetermined mechanism.

### 1.3 AQP2

AQP2 is a water-selective AQP predominantly expressed in the kidney collecting duct. Relocalisation of AQP2 to the apical membrane of kidney collecting duct cells in response to the anti-diuretic hormone, arginine vasopression, was the first discovered example of an AQP regulated by subcellular relocalisation [43], [44], and remains the most well characterised. AQP2 relocalisation has been extensively reviewed elsewhere [45], [46], [47], so we intend to give the reader only a brief overview.

Phosphorylation of AQP2 at Ser256 by PKA appears to be the major determinant of AQP2 localization and is a requirement for vasopressin-stimulated relocalisation of AQP2 from storage vesicles to the apical membrane. Following vasopressin stimulation of rat renal tissue, AQP2 phosphorylation was increased at Ser256, measured by ^32^P labelling and immunoblotting with a phosphor-Ser256-specific antibody [48]. Analysis of the stoichiometry of phospho-Ser256 AQP2 in *Xenopus* oocytes using phosphomimetic (Ser256Asp) and non-phosphorylatable (Ser256Ala) mutants suggested a requirement of at least three phosphorylated monomers per tetramer for apical membrane localization [49]. This PKA-dependent Ser256 phosphorylation has also been implicated in a direct increase in single-channel water permeability. Both cAMP stimulation of AQP2-expressing *Xenopus* oocytes and in vitro phosphorylation of recombinant AQP2 reconstitued into proteliposomes enhanced water permeability [50], [51]. Although the effect in oocytes may be partially explained by relocalization of AQP2 to oocyte membrane, the proteoliposome experiments suggest a direct effect. Despite the important role of Ser256 phosphorylation, there are several other phosphorylation sites in the AQP2 C-terminus that may fine-tune the AQP2 membrane abundance, with subsequent phosphorylation at Ser269 enhancing apical membrane retention [52], [53]. AQP2 phosphorylation at Ser261 and Ser264 were also decreased [54] and increased [55], respectively, by vasopression stimulation of Brattleboro (vasopressin-null) rats. Their roles in AQP2 relocalisation are less well understood but phosphorylation of Ser261 has been associated with localisation in storage vesicles [54], [56], [57] while Ser264 has been proposed to play a role in exosome excretion [55], [58].

Anchoring of PKA by A-kinase-anchoring proteins (AKAPs) to AQP2-containing vesicles has been suggested as a further regulatory mechanism. AKAP220 bound to AQP2 in a yeast two-hybrid screen, and overexpression of AKAP220 in the African green monkey COS-7 cell line increased forskolin-induced phosphorylation of AQP2 [59]. AKAP18δ was present on immunoprecipitated AQP2-containing vesicles from rat kidney tissue, as well as relocalising to the plasma membrane of cultured primary rat inner medullary collecting duct cells in response to vasopressin [60]. Broad spectrum AKAP inhibitors increased AQP2 phosphorylation and membrane localization independently of vasopressin in the mouse collecting duct cell line mpkCCDc14 [61].

During the trafficking process, AQP2 interacts with a number of cellular proteins in a phosphorylation-dependent manner including the actin filaments [62]. AQP2 bound to G-actin in in vitro pulldown experiments, and in live transfected Madin-Darby canine kidney (MDCK) cells measured by fluorescence cross-correlation spectroscopy. The affinity of this interaction, measured by surface plasmon resonance, was decreased by AQP2 phosphorylation. Phosphorylated AQP2 bound to the actin filament-stabilising protein tropomysin-5b (TM5b) in similar experiments, and in an in vitro actin filament stability assay, addition of phosphorylated AQP2 prevented the stabilising effect of TM5b on actin filaments, suggesting that phosphorylation of AQP2 indirectly affects actin depolymerisation by sequestering TM5b [63]. Together, these data suggest that phosphorlyation of AQP2 alters local actin dynamics by altering the concentrations of G-actin and TM5b, in order to locally disrupt the cortical actin network and provide a path to the membrane for AQP2-bearing vesicles. Recently it has been suggested that the Arp2/3 complex, which generates nucleation sites for actin filament branching, is required for AQP2 exit from the trans-Golgi network [64], [65], but the mechanism is not yet understood.

To summarise, the vasopressin-induced relocalisation of AQP2 to the collecting duct apical membrane is dependent on phosphorylation at Ser256 by PKA, which is likely tethered to AQP2-containing vesicles by AKAPs, and requires local modulation of cortical actin dynamics.

### 1.4 AQP3

AQP3 is expressed in the kidney, digestive tract, erythrocytes, lymphocytes, macrophages, dendritic cells, and the skin [66], [67]. When expressed in polarized epithelia, AQP3 is primarily localised to the basolateral membrane. In the canine kidney epithelial cell line MDCK, this is mediated by a tyrosine-based and dileucine sorting motif (Tyr-Arg-Leu-Leu, YRLL) [68]. Although both AQP3 and AQP4 are localised to the basolateral membrane, they appear to be delivered to the plasma membrane by mutually-exclusive post-Golgi vesicle pools [69].

Forskolin treatment of the prostate cancer cell line PC-3 led to internalisation of AQP3, whereas overexpression of E-cadherin or knockdown of the Ras superfamily small GTPase RalA led to translocation of AQP3 to the plasma membrane [70], although whether the effect of forskolin was via RalA or direct PKA phosphorylation of either AQP3 itself or proteins involved in its trafficking is not clear.

In the human colorectal cancer cell line Caco-2, AQP3 was relocalised to the plasma membrane in response to adrenaline. This was blocked by inhibitors of protein kinase C (PKC) and phospholipase C (PLC), and reproduced by the PKC activator PMA, suggesting a Gq/PLC/PKC signal transduction pathway [71]. Whether AQP3 itself was the direct target of PKC was not investigated.

In the mouse 3 T3-L1 cell line differentiated into adipocytes, AQP3 was relocalised to the plasma membrane in response to the β-adrenergic agonist isoproterenol with no effect on AQP3 gene expression. This relocalisation was prevented by inhibition of phosphatidylinositol 3-kinase (PI3K) inhibition with wortmannin or mammalian target of rapamycin (mTOR) inhibition with rapamycin [72].

Overall, the subcellular localisation of AQP3 appears to be under the control of several signalling pathways including PKA, PKC, and PI3K/Akt/mTOR, but the molecular details are yet to be investigated.

### 1.5 AQP4

AQP4 is expressed in astrocytes in the central nervous system (CNS), the kidney, skeletal muscle, and in the digestive tract. AQP4 has a distinct basolateral localisation in many types of epithelial cell, including in the renal proximal tubule, the trachea, and in parietal cells of the stomach [73], [74]. AQP4 membrane expression in astrocytes is polarized to the endfeet (astrocyte processes in contact with the endothelial or pericyte basement membrane at the blood-brain barrier; BBB) and to a subset of astrocyte processes (that form tripartite synapses [75]). In the endfoot membrane, AQP4 can aggregate into large supramolecular arrays, known as orthogonal arrays of particles (OAPs) [76]. The two major AQP4 isoforms (M1 and M23, named from the position of the initiating methionine residue) differ in their OAP-forming ability, with two palmitoylated cysteine residues in the N-terminus of M1 sterically hindering OAP formation [77].

The regulation of AQP4 has been well-studied. In *Xenopus* oocytes transfected with rat AQP4, the AQP4-dependent water permeability was decreased by PKC activators, and ^32^P labelling of mouse brain lysate in the presence of PKC activators suggested that AQP4 was phosphorylated downstream of PKC activation (although this experiment was not designed to distinguish between direct or indirect phosphorylation by PKC). This study did not investigate the subcellular localisation of AQP4 [78]. Vasopressin stimulation of *Xenopus* oocytes co-transfected with rat AQP4-M23 and the human V1a vasopressin receptor (V1aR) led to internalisation of AQP4, which was recapitulated with the PKC activator phorbol 12-myristate 13-acetate (PMA). Mutation of the putative PKC site Ser180 to alanine only partially reduced this response [79], suggesting the involvement of either other PKC sites on AQP4, or other PKC target proteins. In the porcine kidney epithelial cell line LLC-PK1, both dopamine and the PKC activator PDBu slightly reduced plasma membrane water permeability following transfection of mouse GFP-AQP4-M23. This effect was abolished after Ser180Ala mutation, suggesting a role for phosphorylation of Ser180 by PKC in this effect, although quantification of AQP4 plasma membrane localisation was not shown in this study [80]. Molecular dynamics simulation of rat AQP4 did not find any differences in water permeability between p-Ser180 and non-phosphorylated AQP4 [81], although this study relied on an in silico prediction of the structure of the 69aa C-terminal tail of AQP4 with no experimental validation.

In HGT-1 human gastric cancer cells, histamine stimulation induced internalisation of transfected rat VSV-AQP1-M1, which was followed by PKA-mediated phosphorylation of AQP4. This phosphorylation appeared to prime AQP4 for return to the membrane upon washout of histamine [82]. In AQP4-transfected MDCK cell lysates, recombinant casein kinase (CK)II could phosphorylate AQP4, and the phosphomimetic Ser276Asp mutation increased the rate of protein degradation, but not internalisation, with the Ser276Asp protein targeted to the lysosome [83].

The membrane water permeability of an SV40-immortalised rat astrocyte cell line transfected with mouse GFP-AQP4-M23 was increased in response to treatment with lead in a manner dependent on Ca^2+^/CaM-dependent protein kinase (CaMK) II, which the authors proposed as an explanation for brain edema following acute lead intoxication [84]. The same group later demonstrated that glutamate treatment of primary rat astrocytes, rat hippocampal slices, or immortalised astrocytes transfected with mouse GFP-AQP4-M23, increased membrane water permeability by 40%. Mutation of Ser111 to alanine abolished the response to glutamate, although the basal level of water permeability was considerably increased for the Ser111Ala mutant. PKG but not CaMKII was able to phosphorylate a 28-residue mouse AQP4 peptide centred on Ser111, which was abolished by Ser111Ala mutation, suggesting a CaMKII-PKG signalling pathway [85]. AQP4 plasma membrane localisation was not quantified in these studies, and molecular dynamics simulation of human AQP4 found no permeability differences between phosphoSer111 and non-phosphorylated AQP4 [86], suggesting that changes in AQP4 localisation could explain these data. Treatment of rat astrocytes with manganese increased the plasma membrane abundance of AQP4 without affecting AQP4 mRNA or protein levels in a manner dependent on extracellular signal-regulated kinase (ERK)1/2 and p38-MAPK activation [87]. A minor reduction in extracellular pH from 7.5 to 7.1 by either lactic acid, hydrochloric acid, or acetic acid increased the plasma membrane abundance of endogenous AQP4 in primary rat astrocytes by 3-fold after 12 h [88]. This highlights the need for careful cell culture, as such pH changes can easily happen in cell culture experiments if medium is not replenished promptly (medium pH will decrease over time as a result of cell metabolism) or care is not taken with vehicle controls for drugs dissolved in strong acid/base.

AQP4 contains a consensus PDZ-binding motif (SerSerVal) at the C-terminus, and knockout of the PDZ-containing adaptor protein α-syntrophin reduced the BBB polarization of AQP4 in mouse brain [89], as did knockout of dystrophins [90], and the specific dystrophin isoform Dp71 [91]. Dystrophins and syntrophins are key components of the dystrophin-associated protein complex (DAPC), a multi-component membrane scaffolding complex that is crucial for anchoring membrane proteins to the extracellular matrix (ECM) at the BBB [92]. Several extracellular proteins reduced the rate of internalisation of endogenous AQP4 in cultured rodent astrocytes, including agrin [93], and laminin. In the case of laminin, this may be due to induction of preferential binding of the DAPC to inactive dynamin, locally reducing the rate of clathrin-mediated endocytosis [94].

We recently found that plasma membrane localisation of AQP4 in astrocytes was increased by cell swelling induced by either extracellular hypotonicity or hypoxia, and that this was dependent on TRP channel activation, PKA phosphorylation of AQP4 at Ser276 and direct binding of CaM to an amphipathic helix in the AQP4 C-terminal tail, in both primary human astrocytes and HEK293 cells [6], and independent of total AQP4 expression [6], [95]. Interestingly, several other AQPs appear to be trafficked in a calcium-dependent manner but by unknown molecular mechanisms (see section above on AQP1 and below on AQP5), and both AQP0 [20] and AQP6 [96] have been shown to bind CaM directly (leading to channel gating in the case of AQP0), suggesting a possible role for CaM in the regulation of other AQPs. There is also clear cell-type (and possibly species) differences in AQP4 membrane localisation, as it has previously been shown that in MDCK cells, phosphorylation at Ser276 had no effect on AQP4 plasma membrane abundance, and targeted the protein to the lysosome [83].

Overall, AQP4 subcellular localization can be modulated by several protein kinases (PKC, PKA, PKG, ERK, p38-MAPK), by activation of TRP channels, by indirect interaction with the ECM, and by direct binding of CaM. Some studies have suggested that phosphorylation can alter the single-channel permeability of AQP4, but molecular dynamics simulations do not support this hypothesis.

### 1.6 AQP5

AQP5 is primarily expressed in the sweat, lacrimal, and salivary glands, and in the lungs. Loss of AQP5 expression or membrane localisation is associated with dry mouth and dry eye, especially in the autoimmune condition Sjögren's syndrome [97].

Acetylcholine treatment of rat parotid (salivary gland) tissue was reported to cause a transient increase in AQP5 apical membrane localisation, signalling through the M3 muscarinic receptor, and depending on calcium release from intracellular stores [98]. Similarly, adrenaline caused a transient increase in AQP5 apical membrane localisation in rat parotid tissue, signalling through the α1 acetylcholine receptor [99]. In human salivary gland cells transfected with rat AQP5, increased AQP5 membrane localisation was reported following elevation of intracellular calcium with the sarco/endoplasmic reticulum Ca^2+^-ATPase inhibitor thapsigargin, or the calcium ionophore A-23187. This relocalisation was blocked by the microtubule polymerisation inhibitors colchicine and vinblastine [100].

AQP5 was reported to co-localize with the caveolae-associated protein flotillin-2 in rat parotid gland tissue, with flotillin-2 also relocalising to the plasma membrane in response to an α1 receptor agonist [101]. In parotid cells isolated from caveolin-1 knockout mice, AQP5 was unable to relocalise in response to α1 receptor agonist, attributed to a loss of interaction between TRPC1, the calcium-sensor STIM1, and the calcium release-activated calcium channel ORAI1. It is not yet clear exactly how elevated intracellular calcium ion levels lead to membrane trafficking of AQP5. Interestingly, we found a role for the calcium ion-sensor protein CaM in the hypotonicity-induced relocalisation of AQP1 [37] and AQP4 [102], and recently showed that CaM binds directly to the AQP4 C-terminus [6]. Both AQP0 [20] and AQP6 [96] can bind CaM directly, so this may be an unexplored mechanism by which AQP5 is targeted to the plasma membrane.

We reported that hypotonicity, phosphomimetic mutation of Ser156 (Ser156Glu) and PKA inhibition with H-89 all increased plasma membrane localisation of human AQP5-GFP in HEK293 cells independently of one another, suggesting that there are at least three distinct pathways controlling AQP5 membrane localisation in HEK293 cells [103]. In agreement with our study, PKA inhibition by H-89 in MDCK cells transfected with rat GFP-AQP5 caused increased apical membrane localisation [104]. Interestingly, an almost identical study, again using rat GFP-AQP5 transfected into MDCK cells, found an opposite effect – that activation of PKA with dibutyryl-cAMP caused no short-term change in AQP5 membrane abundance, but a long-term (18–24 h) increased membrane abundance of AQP5, that could be inhibited with H-89 [105]. The latter study used subconfluent MDCK cells cultured on glass (which would not be expected to develop apicobasal polarity), whereas the former study used confluent MDCK cells on polycarbonate filters (MDCK cells develop apicobasal polarity under these conditions) and specifically measured apical membrane localisation. In agreement with this long-term increase, AQP5 membrane abundance in cultured mouse and human airway epithelial cells was increased at 8 and 24 h after 8-(4-chlorophenylthio)-cAMP (pCPT-cAMP) stimulation, but decreased after 30 min [106], [107]. This latter finding agrees with the aforementioned studies that found increased AQP5 membrane abundance following PKA inhibition.

Experiments using rat parotid gland tissue slices found that hypotonicity and the α1-adrenergic agonist phenylephrine increased AQP5 apical membrane abundance independently of one another, with only the hypotonicity-induced increase (and not the α1-adrenergic pathway) abolished by the TRP channel blocker ruthenium red [108], suggesting a requirement for TRP channel activity in the hypotonicity-induced relocalisation of AQP5, similarly to AQP1 in HEK293 cells [37] and AQP4 in primary human astrocytes [6].

Overall, AQP5 subcellular localization can be modulated by PKA, by activation of TRP channels, and by intracellular calcium signalling.

### 1.7 AQP6

AQP6 is expressed in renal epithelia and localizes to intracellular membranes [109]. Unlike other aquaporins, AQP6 is an anion transporter (transporting NO_3_^−^, I^−^, Br^−^, and Cl^−^ [110]), that can be converted into a water conducting pore by a single amino acid substitution [111], and the permeability to both water and anions of mouse AQP6 were increased by low pH [112]. However, it is still unclear whether the main function of AQP6 is transport of water, anions or both. Although the molecular mechanisms of AQP6 intracellular retention are not completely understood, addition of GFP or HA tags to the N-terminus of rat AQP6 caused plasma membrane localisation in confluent MDCK cells, whereas C-terminally tagged and untagged AQP6 were retained in intracellular membranes. A chimera protein containing the N-terminus of AQP1 fused to the transmembrane and C-terminal domains of AQP6 localised to the plasma membrane, and the inverse chimera containing the N-terminus of AQP6 fused to AQP1 was retained intracellularly whereas wild-type AQP1 was localised to the plasma membrane [113]. Together this data suggests the presence of an intracellular retention signal in the AQP6 N-terminus. In *Xenopus* oocytes, rat AQP6 is localised to the plasma membrane [114], suggesting cell- or species-specific differences in intracellular retention.

Overall, although it is established that AQP6 can transport anions and localize to intracellular membranes, the physiology and molecular biology of this remains almost completely unexplored.

### 1.8 AQP7

AQP7 is an aquaglyceroporin, permeable to water, glycerol and urea [115]. AQP7 is expressed in various organs including liver, kidney, male reproductive system, and cardiac muscle [116]. Additionally, it was shown to be expressed in murine and human white and brown adipose tissues, where it was revealed to play a significant role in glycerol efflux [117].

Knockout of AQP7 in mice has been shown to result in adipocyte hypertrophy leading to adult-onset obesity [118], likely due to increased glycerol and triglyceride accumulation [116]. This effect is not seen in humans with AQP7 loss-of-function mutation [119], probably due to the ability of adipocyte AQP10 to compensate for the loss of AQP7 (*Aqp10* is a pseudogene in mouse) [120]. In mouse adipose tissue, norepinephrine stimulation as well as forskolin were reported to cause internalisation of AQP7 [121], and live cell imaging of 3 T3-L1 adipocytes transfected with AQP7-EGFP supported this conclusion. Interestingly, this study found considerable differences in AQP7 subcellular localization when tissue was fixed with trichloroacetic acid compared to formaldehyde. Fixation methods are rarely compared in studies of AQP localization; these data indicate that this is a key experimental detail to consider. In contrast, short-term treatment of primary human adipocytes with isoproterenol was reported to cause translocation of AQP7 to the plasma membrane without changes in expression, whereas long-term stimulation also reduced protein expression [72]. In agreement with this finding, AQP7 was reported to translocate to the plasma membrane after 3 h isoproterenol treatment in 3 T3-L1 adipocytes, measured by immunocytochemistry [122].

AQP7 has been shown to bind perilipin 1 (PLIN1), a lipid droplet surface protein that has been shown to protects droplets from lipase activity [123]. Recombinant human AQP7 expressed in *Pichia pastoris* was able to pull down PLIN1 from human adipose tissue lysate, and in dot blots, purified AQP7 was able to bind PLIN1 from PLIN-1 expressing E.coli cell lysate in a manner dependent on both the N- and C-termini of AQP7. Recombinant PKA was able to phosphorylate recombinant AQP7, with the S10A/T11A double mutation abolishing phosphorylation, and phosphorylated AQP7 bound less PLIN1 in dot blots. AQP7 and PLIN1 were colocalised in primary human astrocytes as shown by proximity ligation assay, with the extent of colocalization reduced following lipolytic (isoprenaline) stimulation [124]. This suggests a model of AQP7 regulation whereby phosphorylation of AQP7 in the N-terminus by PKA releases AQP7 from PLIN1-mediated tethering to the lipid droplets, allowing translocation to the plasma membrane.

In summary, AQP7 subcellular localization in adipocytes is under the control of lipolytic stimuli via PKA, and facilitates the release of lipolysis-derived glycerol.

### 1.9 AQP8

AQP8 can be localised to both plasma and intracellular membranes, including the inner mitochondrial membrane [125]. It is expressed in kidney, liver, pancreas, colon, trachea and testis [126], and is permeable to water, ammonia, hydrogen peroxide, and possibly urea, although there may be species-specific differences in urea permeability due to differences in pore-lining residues [127].

AQP8 subcellular localisation has been studied most extensively in hepatocytes, where it may be important for bile formation. In isolated primary rat hepatocytes, approximately 25% of the total AQP8 pool was localised to the plasma membrane under basal (unstimulated) conditions, measured by immunofluorescence and membrane fractionation by density gradient centrifugation, approximately doubling to 60% following stimulation with dibutyryl-cAMP in one study [128], and increasing by approximately fourfold in another [129]. This effect was recapitulated by stimulation with glucagon, a known inducer of bile secretion, which also increased plasma membrane water permeability in proportion to the increased AQP8 plasma membrane localisation [130]. Relocalisation of AQP8 was prevented by inhibition of PKA with H-89 or myristoylated protein kinase inhibitor peptide (myr-PKI) [130], and by inhibition of phosphoinositide 3-kinase (PI3K) with wortmannin or LY294002 [131]. However, the exact mechanism remains unclear since AQP8 lacks a consensus PKA phosphorylation site [132].

To summarise, AQP8 localisation in the liver is under the control of PKA and PI3K signalling, but its subcellular localisation in other tissues remains largely unexplored.

### 1.10 AQP9

AQP9, an aquaglyceroporin, is expressed in hepatocytes, epididymal cells [133] and several types of immune cell such as neutrophils, macrophages and lymphocytes [67]. In the rodent CNS, AQP9 is expressed in astrocytes, catecholaminergic neurons and endothelial cells of pial vessels [134], [135], although CNS expression may be more restricted in humans [136]. AQP9 is permeable to glycerol and lactic acid [137], and thus it has been hypothesized that it has a role in brain energy metabolism in addition to water homeostasis [138]. In murine primary hepatocyte culture, AQP9 knockout reduced the glucose output from the liver when using glycerol as a substrate, suggesting that AQP9 facilitates glycerol uptake for gluconeogenesis [139], [140]. In perfused rat epididymal tubules, AQP9-dependent glycerol-induced epididymal cell swelling was regulated by cAMP, although the role of AQP9 localization in this regulation was not explored [141].

In mouse neutrophils and the human HL60 acute promyleocytic leukaemia cell line, exposure to a chemotactic gradient caused human EGFP-AQP9 to translocate to the leading edge of the cell, and Ser11Ala mutation prevented this translocation [142]. AQP9 phosphorylation was induced by the PKC activator PMA, and recombinant PKC was able to phosphorylate EGFP-AQP9 in vitro, with a stronger ^32^P signal for wild-type AQP9 compared to the Ser11Ala mutant [143]. Altogether, this suggests that AQP9 translocation in neutrophils is mediated by direct PKC phosphorylation at Ser11.

Overall, AQP9 localisation may be regulated by PKA and PKC, but the molecular details remain to be elucidated.

### 1.11 AQP10

AQP10 is an aquaglyceroporin, and is expressed in fat and the gastrointestinal tract. The molecular details of the regulation of lipolysis and glycerol flux are not well understood; however, AQP trafficking is suspected to play a key role [122], [144]. In addition to the highly abundant AQP7 in human adipocytes, AQP10 has been shown as an alternative pathway for glycerol efflux [122]. Interestingly, AQP10 is not expressed in mice where it is a pseudogene [145], which may explain the difference in phenotype between AQP7^−/−^ mice and AQP7 loss of function mutations in humans [122]. Human AQP10 was reported to be glycerol-impermeable at pH 7.4, with glycerol permeability induced by intracellular acidification, mediated by protonation of an intracellular histidine residue (H80), based on experiments with human adipose tissue-derived membrane vesicles and recombinant human AQP10 reconstitued into PDMS-PMOXA polymersomes [146]. In contrast, other studies have reported robust glycerol permeability of AQP10 in human adipocyte-derived membrane vesicles at pH 7.4 [122], *Xenopus* oocytes overexpressing human AQP10 cultured at pH 7.4 [147], proteoliposomes containing recombinant human AQP10 at pH 8.0 [148], and HEK293 cells overexpressing human AQP10-GFP cultured at pH 7.4 [149]. Glycosylation has been shown to affect the thermostability of recombinant human AQP10 [148]. Whether glycosylation has any affect on the subcellular loclaisation of AQP10, or indeed any other AQP, remains to be investigated. Insulin treatment of cultured human adipocytes increased AQP10 (and also AQP7) localisation in the vicinity of lipid droplets, whereas isoproterenol (a β-adrenergic agonist) treatment increased the abundance of AQP10 in the plasma membrane [122]. This suggests a similar trafficking mechanism for AQP10 in adipocytes as previously demonstrated for AQP7 [72] with internalisation driven by lipogenic stimuli, and relocalisation to the plasma membrane driven by lipolytic stimuli.

Some studies have suggested that AQP10 is integrated directly into the lipid droplet membrane. The physicochemical properties of the lipid droplet membrane are considerably different from the plasma membrane and most other intracellular membrane compartments. Whereas most membranes are composed of a phospholipid bilayer with a hydrophilic environment either side, the lipid droplet membrane consists of a phospholipid monolayer with a hydrophilic environment on one side (cytosol) and a hydrophobic environment on the other side (lipid droplet interior). As polytopic transmembrane proteins, aquaporins have hydrophilic intracellular and extracellular domains (i.e. the N- and C-termini and intra- and extracellular loops). It is therefore difficult to imagine how such a protein could be stably inserted into the lipid droplet membrane. In our view it is more likely that AQP10 is localised to the membranes of vesicles in the vicinity of the lipid droplet. Immunofluorescence data support this view (see for example Fig. 4 in Miyauchi et al. [121]), although revisiting this question using new developments in super-resolution or expansion microscopy could help to unequivocally resolve this. Exactly how localisation of an aquaglyceroporin to peri-droplet vesicles would alter lipid droplet glycerol dynamics is unclear. One possibility is that by sequestering released glycerol into peri-droplet vesicles, the concentration gradient for glycerol diffusion out of the lipid droplet is maintained. Alternatively, it may simply be that peri-droplet localisation sequesters the AQP away from the plasma membrane and recycling pools.

To summarise, AQP10 localisation in adipocytes is under the control of lipolytic and lipogenic stimuli, but the molecular details remain to be established, and the subcellular localisation of AQP10 in other tissues is not well understood.

### 1.12 AQP11

AQP11, along with AQP12 form a sub-family of ‘superaquaporins’ that display very low sequence homology to other aquaporins and are primarily localised to the membranes of intracellular organelles instead of the plasma membrane [150].

AQP11 is localised primarily to the endoplasmic reticulum (ER) membrane, although a small proportion of the AQP11 pool may reach the plasma membrane in some cell types [151], [152]. It is hydrogen peroxide permeable [152], [153], and may be water and glycerol permeable [152], although different expression systems give conflicting results [154]. AQP11 is expressed in kidney, liver, testes, brain, and fat [152], [154], and AQP11 knockout mice die 1 month after birth with polycystic kidneys, possibly caused by ER dysfunction [155]. AQP11 is localised to the plasma membrane when overexpressed in *Xenopus* oocytes [154], suggesting that the intracellular retention signal(s) may not be active in these cells. Little is known about the molecular basis of the intracellular retention or plasma membrane trafficking of AQP11.

### 1.13 AQP12

AQP12 is expressed in the intestine, pancreas, stomach, and tongue in human, mouse, rat and chicken as well as in the pancreas for all species except rat [156]. Under normal conditions AQP12 localizes to the basal side of the intracellular organelles of pancreatic acinar cells, mainly to the rough ER membrane, and to the zymogen granules near the ER. Following careluein-induced acute pancreatitis rat AQP12 was localised more to the apical side of the cells [157]. The trafficking, localization, function, and regulation of AQP12 remain largely unclear.

## 2 Summary

Regulation of AQP function by subcellular relocalisation is a ubiquitous regulatory mechanism across the mammalian AQP family. This phenomenon is relatively well-studied for AQP0–5, whereas there is comparatively little information available in the literature on subcellular relocalisation of AQP6–12. Given that the study of AQP0–5 relocalisation has led to identification of many components of the underlying molecular machinery and hence new drug targets, we encourage the field to further investigate the subcellular localisation of AQP6–12, which may lead to the identification of novel drug targets for pathologies associated with these AQPs.

Although many AQPs in a variety of cell types have been shown to either translocate to the plasma membrane or be internalised upon kinase activation, it is often not clear whether the AQP is the direct target of the kinase. We therefore propose that when AQP localisation experiments indicate the involvement of a kinase, they should be routinely supplemented with experiments using phospho-proteomic approaches, mutation of putative kinase sites on the AQP, in vitro phosphorylation assays with purified kinase, or phospho-specific antibodies.

We also note that many studies of AQP subcellular localisation are done using an AQP cDNA from one species transfected into cells of another species (e.g. rat AQP4 transfected into human cells, or AQPs from a variety of species transfected into the canine MDCK cell line). There are species-specific differences in AQP sequences which may impact localisation and function. For example, phosphorylation of AQP4 at Ser315 can be detected by mass spectrometry of rat [158] and mouse [159] brain samples, but this phosphorylation site is not conserved in human AQP4 (residue 315 is a glutamine). Therefore, studies in which rodent AQP4 is transfected into human cells is subject to potential artefacts due to phosphorylation at Ser315 which is not possible for human APQ4. We strongly recommend that AQP localisation studies are done using cDNA clones from the same species as the expression system, and that where possible, key findings are replicated for the endogenous AQP in appropriate primary cells or tissue.

Various AQPs have polarized distribution in vivo that can be difficult to accurately reproduce in culture. Recent advances in 3-dimensional cell culture, in particular organoid and organ-on-a-chip co-culture models, may go some way towards bridging the gap between simple 2-dimensional cell monocultures and the complex 3-dimensional organisation of real tissues [160], [161]. How AQP *function* might be quantified in such models is a challenging problem that is yet to be addressed.

We have recently shown that CaM binds AQP4, and CaM can also bind to both AQP0 and AQP6. Several other AQPs are also relocalised in a calcium and/or CaM-dependent manner (AQP1, AQP5), but by an unknown molecular mechanism, suggesting that CaM binding may be a mechanism by which AQP function can be coupled to calcium signalling across the AQP family more broadly.

AQPs support a wide range of physiological and pathophysiological processes. Yet, there is no water-channel-blocking drugs for any AQP have been approved for use in humans [162], [163]. Understanding their regulation is likely to lead to new drug targets for a variety of pathologies [164], [165], as well improving understanding of cellular, organ, and organism-level water and solute homeostasis.

## Funding

This work was supported by 10.13039/501100004950Aston University through a 50th Anniversary Prize fellowship to PK and a PhD studentship to AM. RMB, ACC and PK were supported by the Biotechnology & Biosciences Research Council (BB/P025927/1). LU is supported by the 10.13039/501100000780European Union‘s 10.13039/100010661Horizon 2020 research and innovation programme under Marie Skłodowska Curie grant agreement No. 847,419 (MemTrain). MA is supported by a studentship co-funded by 10.13039/501100004950Aston University and the 10.13039/501100000266UK Engineering and Physical Sciences Research Council (EP/R512889/1) to RMB. AS is supported by a studentship from the 10.13039/501100007335Iraqi Ministry of Higher Education and Scientific Research and the University of Mosul. AH was supported by a studentship from 10.13039/501100007922Spinal Research. STH is supported by the 10.13039/501100004359Swedish Research Council (2013-05945), the 10.13039/501100003173Crafoord Foundation (20140811 and 20180916) and the 10.13039/501100006285Magnus Bergvall Foundation (2015-01534).

## Declaration of competing interest

The authors declare that they have no known competing financial interests or personal relationships that could have appeared to influence the work reported in this paper.
